# Anthelmintic resistance of horse strongyle nematodes to ivermectin and pyrantel in Lithuania

**DOI:** 10.1186/s13028-021-00569-z

**Published:** 2021-01-25

**Authors:** Evelina Dauparaitė, Tomas Kupčinskas, Georg von Samson-Himmelstjerna, Saulius Petkevičius

**Affiliations:** 1 Laboratory for Parasitology, Department of Veterinary Pathobiology, Veterinary Academy, Lithuanian University of Health Sciences, Tilzes str. 18, 47181 Kaunas, Lithuania; 2 Institute for Parasitology and Tropical Veterinary Medicine, Free University of Berlin, Robert-von-Ostertag Strasse 7-13, 14163 Berlin, Germany

**Keywords:** Efficacy, Helminths, In vivo, Selective therapy

## Abstract

**Background:**

With intensive use of anthelmintic drugs in recent decades, anthelmintic resistance (AR) in horse nematodes is becoming a growing issue in many countries. However, there is little available information about the parasites, treatment practices or AR in the horse population in Lithuania. The aim of this study was to assess the current situation of AR on horse farms in Lithuania. The study was conducted in 25 stables on horses with a strongyle faecal egg count (FEC) of ≥ 200 eggs per gram. A faecal egg count reduction test (FECRT) was performed on each farm after administration of ivermectin (IVM) or pyrantel (PYR).

**Results:**

The efficacy of IVM was comparatively high, with 98.8% of 250 horses having a zero egg count 14 days after treatment. Two conditions were used to interpret the FECRT results for PYR: firstly, resistance was determined when FECR was < 90% and the lower 95% confidence interval (LCL) was < 80%, and secondly when in addition the upper confidence level (UCL) was < 95%. Under the first condition, resistance against PYR was found in five stables (25% of all tested herds), while when considering the UCL as well, resistance was only detected in two stables (8%). The FEC showed a significant (P < 0.01) difference between the treatment and control groups. Only cyathostomin larvae were detected in larval cultures derived from strongyle-positive faecal samples collected 14 days after treatment of a test group with PYR.

**Conclusions:**

This in vivo study showed that PYR resistance is prevalent on horse farms in Lithuania, while the efficacy of IVM still appears to be unaffected. However, further studies of ivermectin resistance are needed. These findings should guide the implementation of more sustainable management of strongyle infections in horses in Lithuania.

## Background

Strongylid (family Strongylidae) nematodes, such as *Strongylus* (large strongyles) and cyathostomins (small strongyles), are the main internal nematode parasites of horses and constitute more than 75% of the total intestinal parasite fauna [1, 2]. Large strongyles, such as the highly pathogenic *Strongylus vulgaris*, are no longer commonly found in domestic horses [3, 4]. This has led to a shift in the focus of anthelmintic treatment programmes to the less pathogenic, but more common and abundant cyathostomin parasites [5, 6].

The increased resistance of horse nematodes to anthelmintic drugs [7] is a matter of concern all over the world. The use of uncritical anthelmintic treatment strategies rather than evidence-based treatments may partly explain the apparent increase in reports of resistance [5]. Resistance to benzimidazole has been reported all over the world [8–11], while resistance to pyrantel (PYR) is emerging in the USA, UK, Germany and Italy [12–14]. In other European countries close to Lithuania, the situation regarding resistance to benzimidazole and pyrantel is fairly similar. Resistance to benzimidazole has been reported in Poland [15] and Ukraine [16], and resistance to pyrantel has been reported in Estonia [17] and Sweden [18]. The last time resistance to benzimidazole was confirmed in Lithuania was by Vyšniauskas et al. [19]. Due to the appearance of resistance to these two drug classes, in the past two decades great reliance has been placed on macrocyclic lactones (ML). Reports from France [20], Germany [21], Belgium, the Netherlands [22] and the UK [23] have revealed a shortened strongyle egg reappearance period (ERP) after treatment with ivermectin or moxidectin, which is interpreted as an early indication of emerging resistance. The current status of anthelmintic efficacy in domestic horses is of importance for both scientists, veterinarians and horse owners, particularly because no new drug classes or modes of action have been introduced since ivermectin in the 1980s [24].

It is widely accepted that due consideration of the role of parasite *refugia* is key to preserving the efficacy of anthelmintic drugs in worm control programmes [25]. One way to maximise *refugia* is to apply selective targeted treatment as part of a sustainable equine nematode control programme [26]. For worm control in adult horses, guidelines from the European Scientific Counsel Companion Animal Parasites (ESCCAP) and the American Association of Equine Practitioners (AAEP) recommend using FEC for surveillance, based on anthelmintic treatment programmes in which only those horses above an arbitrary cut-off of typically ≥ 200 eggs per gram (FEC) are treated [6, 27]. Alternatively, for adult horses where selective treatments are not feasible and particularly concerning worm control in young horses, ESCCAP guidelines for strategic treatments aim to reduce treatment frequencies and vary the drug classes [27]. To ensure this can happen, it is important to have information on the actual drug efficacy, which should be regularly assessed on each farm by post-treatment efficacy testing.

The widely used test to assess anthelmintic efficacy is the *in vivo* FECRT. The World Association for the Advancement of Veterinary Parasitology (WAAVP) has issued guidelines for the procedure and calculation of FECRT [28]. However, there is no single agreed interpretation of the outcome of FECRT and different recommendations have been made to classify drug efficacy as “resistance” or “susceptible”, thus the results obtained in different studies cannot always be compared directly [7]. WAAVP’s current recommendations advise considering the presence of resistance if, firstly, the FECR is < 90% and secondly the 95% lower confidence level (LCL) is < 90%. Resistance is deemed ‘possible’ if one of the two conditions is met. This accounts for BZ and IVM in sheep and goats. For BZ in horses, the cut-off point is set at FECR <90% but no LCL value is given [28]. More recently, Lyndal-Murphy et al. [29] have shown that it is important to include the 95% upper confidence limit (UCL) when classifying FECRT results and that the presence of resistance can only be confirmed if the UCL is < 95%.

Lithuania is estimated to have a horse population of about 15,800 individuals (Lithuanian Animal Registry Centre) and these horses travel widely. However, there is little information in the Baltic region about anthelmintic resistance (AR). There have been very few equine parasitology surveys conducted in Lithuania—the last study involving equine AR in Lithuania was published more than fourteen years ago [19]. As a result, very little is known about the anthelmintic efficacy status of the various formulations available. Therefore, the aim of this study was to provide further information about the observed efficacy of frequently used formulations of ivermectin and pyrantel against populations of strongylid parasites on horse farms in Lithuania.

## Methods

The study was carried out between April and November 2019 in twenty-five stables in the northern (44%), central (28%), western (16%) and eastern (12%) regions of Lithuania. Horses on these farms are used for sport, leisure riding and breeding.

In order to find comparable results, larger stud farms, i.e. with at least 35 horses, were pre-selected from the database of the Lithuanian Equestrian Federation (LEF) [30]. The initial screening included 707 horses, and further studies were performed on the horses that met the inclusion criteria, which were strongyle faecal egg counts exceeding 200 eggs per gram (EPG). All horses had access to pasture and had not received any antiparasitic treatment in the eight weeks prior to the study. A total of 659 horses met these inclusion criteria.

Faecal samples were analysed quantitatively using a modified McMaster technique with a detection threshold of 20 EPG [31]. Fresh faecal samples were collected from a pen containing one horse within one hour of defecation or by rectal extraction, and these were stored at 4 °C and processed within 24 h.

Faecal egg count reduction tests (FECRT) were performed on the 659 horses that met the inclusion criteria. The efficacy of both drugs was evaluated in accordance with the recommendations of the World Association for the Advancement of Veterinary Parasitology (WAAVP). Anthelmintic resistance in horses was determined as the percentage reduction of mean egg counts at 14 days post-treatment: FECR% = 100 × (1–(X1/X2)(Y1/Y2), where X1 and X2 represent the mean pre-treatment and post-treatment faecal nematode egg counts (FECs) of a treated group, and Y1 and Y2 represent the mean pre-treatment and post-treatment FECs of an untreated control group respectively [28]. On each farm, the horses selected for testing were randomly assigned to three experimental groups: group A was treated with IVM (n = 10), group B with PYR (n = 10) and group C was the untreated control group (n ≥ 5). The numbers of animals in the respective treatment groups were based on the recommendation that 10 animals per group are considered sufficient to detect differences in FEC between groups [28].

The weight of each animal was estimated using a girth measuring tape. The anthelmintic dosages and routes of application were in accordance with the drug manufacturers’ recommendations. IVM (0.2 mg per kg body weight) was administered *per os* using the product Bimectin^®^ (Cross Vetpharm Limited, Ireland) and PYR embonate (19 mg per kg body weight) was administered *per os* using the product Embotape^®^ (Cross Vetpharm Limited, Ireland).

### FECR was classified using two different sets of methods

Assessment method 1 classified the results as “no resistance”, “suspected resistance” and “confirmed resistance” using the observed FECR and LCL, according to the WAAVP recommendations [28], which for cattle considers FECR of < 95% with a 95% LCL of < 90% as “confirmed resistance”, while a result where only one of the two criteria is met is considered as “suspected resistance”. These recommendations propose a value of < 90% as being indicative of resistance in horses, but give no value for the 95% LCL. Hence, in line with other recent equine AR studies [13, 32], the cut off was set at 90% FECR and the 95% LCL was set at 80% in this study.

Assessment method 2 used a method similar to that advocated by Lyndal-Murphy et al. [29], classifying the parasites as “resistant”, “inconclusive” or “susceptible” based on the following conditions: they were “resistant” if the observed FECR is below 90%, LCL is below 80% and UCL is below 95%, “susceptible” if the observed FECR is at/above 90% and LCL is at/above 80%, and “inconclusive” if none of the conditions are met.

### Differentiation of third-stage larvae

Faecal samples collected on each farm on day 14 were pooled and processed for coproculture. A minimum of 3 g from each strongyle-positive sample were mixed together and incubated for 7 days at room temperature in the laboratory (24–29 °C) (adding water to maintain an adequate moisture level and 4 g of vermiculite). Third-stage larvae (L_3_) were subsequently recovered from the coprocultures using the Baermann technique [33]. The L_3_ larvae were microscopically examined, differentiated by morphology characteristics, and identified according to Maff [34]. The first 100 L_3_ larvae, or all L_3_ when ≤ 100 developed L_3_ larvae, were identified per sample. They were identified by the number, shape and arrangement of intestinal cells [35].

### Statistical analysis

Data were analysed using the statistical software package “SPSS for Windows version 20” and descriptive statistics (means, standard deviations, reduction percentages etc.) were calculated. ANOVA was used to compare the mean EPG of each experimental group. Calculation of the arithmetic mean, the percentage of reduction, the 95% upper and lower confidence limits, and interpretation of the findings was as described by Coles et al. [28]. A probability (P) value less than 0.05 was used to determine the level of significance.

## Results

A total number of 707 horses from 25 farms in Lithuania were coprologically examined in 2019. Of these, 659 horses were used to determine prevalences.

The mean pre-treatment and post-treatment FEC, the FECR percentage and the lower and upper 95 % confidence limits for each group of anthelmintic drugs tested are summarised in Additional file 1.

On three of the 25 farms (12%), the FECR in the IVM and PYR groups was 100%.

### Faecal egg count reduction test with ivermectin

A total of 250 horses were treated with IVM and 156 horses were left untreated as a control group. On 22 of the 25 farms (88%), the FECR was 100% after treatment, and all the horses in this treatment group had a zero egg count on the second visit. Three farms had at least one horse with positive egg count of 20 EPG on day 14. The FECR on these farms was 99.9% (LCL 99.5%), 99.9% (LCL 99.7%) and 99.7% (LCL 98.6%) respectively. IVM-treated horses had significantly (P < 0.01) lower egg counts than the untreated group 14 days after treatment.

### Faecal egg count reduction test with pyrantel embonate

A total of 250 horses were treated with PYR and 156 horses were left untreated as a control group. On three of the 25 farms (12%) the FECR was 100%, and on five farms (20%) the FECR was < 90% (See Additional file 1). PYR-treated horses had a lower egg count (P < 0.01) than the untreated horses 14 days after treatment.

### Interpretation of FECR and 95% CI based on the different assessment methods

With the IVM results assessment by methods 1 and 2, both methods confirmed that ‘no resistance’ to IVM was found on the 25 (100%) farms (Table 1).

**Table 1:** The ivermectin and pyrantel faecal egg count reduction classification using two different sets of methods

Drug class	Method No. 1	Horse stud farms	Method No. 2	Horse stud farms
IVM	Confirmed resistance	0	Resistant	0
	Suspected resistance	0	Susceptible	10
	No resistance	25	Inconclusive	25
PYR	Confirmed resistance	5	Resistant	2
	Suspected resistance	1	Susceptible	16
	No resistance	19	Inconclusive	7

PYR, pyrantel, IVM, ivermectin

With the PYR results assessment by method 1, ‘confirmed resistance’ to PYR was found on five farms (25%), ‘suspected resistance’ was found on one farm (4%), and ‘no resistance’ was found on 19 farms (79%). Assessing the results by method 2, two out of the 25 (8%) farms were classified as ‘resistant’, sixteen farms (64%) as ‘susceptible’, and seven farms (28%) as ‘inconclusive’ (Table 1).

All the third-stage larvae isolated from strongyle egg positive faecal samples were identified as cyathostomin larvae.

## Discussion

Control of equine nematodes has relied on benzimidazoles (BZ), tetrahydropyrimidines and macrocyclic lactones. The intensive use of anthelmintics has led to the development of AR in equine cyathostomins around the world [36]. Monitoring of AR in cyathostomin is one of the most important topics in equine parasitology, but the last study about equine AR in Lithuania was published more than fourteen years ago [19].

Of all the screened candidates (n = 707), 93% exceeded the value of 200 strongyle EPG, which is considered the treatment threshold in most current recommendations concerning selective treatment strategies. One of the basic principles of selective anthelmintic treatment is a consistency of the relative magnitude of strongyle FEC of individual horses over time [37, 38]. Therefore, identification of high egg shedders within the herd is an essential goal [38–40]. It is proposed that the identification of animals regarded as ‘high egg shedders’ enables farms and studs to implement more targeted and/or selective treatment approaches to helminth control [38, 40]. It is known that acquisition of information about natural distribution patterns will help establish appropriate FEC thresholds at which horses should be treated with an effective anthelmintic (commonly quoted as 200–250 EPG) [5, 38].

This would achieve the dual goal of controlling induced health problems while simultaneously reducing pasture contamination [14]. The analysis of the screening FEC data pre-FECRT (not shown) in the present survey demonstrated that there was a higher percentage of “high egg shedders” (i.e. > 200 EPG) than “low or null egg shedders” (i.e. < 200 EPG), thus confirming that current control programmes are not ideal and providing further support for the value of conducting FECs before planning any anthelmintic treatment in a yard. Leaving a proportion of horses untreated would maximise the *refugia* with little impact on overall control, as horses with low egg counts are not important sources of environmental contamination. Additionally, egg shedding from untreated animals would dilute the presence on the pasture of any eggs shed by treated animals possibly infected with resistant populations. In this way, the selection pressure would be progressively reduced [12, 14].

This study demonstrated a very high efficacy of IVM on reducing helminths in horses in Lithuania. As expected, the mean percentage efficacy of IVM in eliminating strongyle eggs from animal faeces ranged from 99.7% to 100% (by FECR) for IVM on all stud farms. On three of the 25 farms, FEC≥20 EPG 14 days after treatment, but the FECR still indicated a high susceptibility to the drug. Comparable results have been described in a Lithuanian report in 2006 [19], where a 100% efficacy of IVM against strongyle was shown in a group of ten study horses. In Estonian investigations, FECR after treating horses with IVM was 100% [17], 100% in Latvia [41], 99.9% in Poland [42] and 99% in Sweden [18]. However, there have been few reports describing incidences of reduced efficacy of IVM against cyathostomin nematodes [14, 43, 44]. Indications of shortened ERP have been found in Germany [21], Belgium, the Netherlands [22] and Finland [45]. Furthermore, findings repeatedly suggest emerging ML resistance in cyathostomins due to reduced ERP following IVM and moxidectin treatments [21, 22, 46]. The shortened ERPs following IVM and moxidectin treatments have been associated with emerging ML resistance by fourth-stage larvae [47]. While resistance to these drugs is still not prevalent, proper use of the drugs *via* selective treatment using FEC and continued monitoring of efficacy with FECRT is of major importance in slowing down the development process of resistance in equine gastrointestinal parasites.

Field studies indicate that PYR resistance is widespread in equine intestinal nematodes in Europe and other regions. Previous studies have already shown that cyathostomin populations resistant to PYR are present in the southern USA, with over 40% of farms demonstrating resistance to this drug [12]. Furthermore, resistance to PYR has been proven in two out of sixteen farms in Italy (12.5%) and suspected in one case (6.2%), with FECR values ranging from 43% to 85.4% [48]. Strongyles have been found to be resistant to pyrantel in Finland (43%), indicating widespread resistance [45]. Geographically near Lithuania, PYR-resistant populations of cyathostomins have been found on Swedish horse farms, but the overall efficacy of PYR is still considered acceptable [18]. Lassen et al. [17] have reported resistance to PYR in Estonia, with FECR of approximately 88% on four horse farms. These results are consistent with those found in this study. Resistance to PYR was detected on five farms (19%), with FECR calculated and interpreted in line with WAAVP recommendations. However, horses from only two of the five farms were still interpreted as having “resistance”, as recommended by Lyndal-Murphy et al. [29]. Cyathostomins were detected in larval cultures derived from strongyle positive faecal samples collected 14 days after treatment with PYR. It should be noted that Pyrantel embonate (European Pharmacopeia) is considered synonymous with pyrantel pamoate (U.S. Pharmacopeia). The label indications of these two pyrantel salts are virtually interchangeable at the same concentrations and dosages. In the USA, pyrantel pamoate is approved as a broad-spectrum equine anthelmintic at a dosage of 6.6 mg/kg. However, certain formulations made by specific sponsors are also labelled for efficacy against equine tapeworms (*Anoplocephala perfoliata*) at a “3X” dosage of 19.8 mg/kg. This number is close to the pyrantel dosage (19 mg/kg) cited in the manuscript, and the product name (Embotape) suggests that this formulation is labelled for efficacy against nematodes and tapeworms in horses. The doses in this study were based on the recommendations of the European Pharmacopoeia and the dosages given in the package leaflet, but a dose of 6.6 mg/kg should be considered in future because the actual pyrantel resistance situation in Lithuania could be significantly higher than that established in the present study.

This in vivo study showed that horse farms in Lithuania are already facing problems with AR, especially resistance to PYR. Despite the fact that resistance was found on five farms and in a small number of horses, an increase in AR can be expected in future. This demonstrates the need for regular parasite and anthelmintic susceptibility monitoring and also the implementation of measures that would delay further development of AR on horse farms.

Infection followed by the development of AR can be controlled by correct administration of anthelmintic substances and a reduction in treatment frequency, e.g*.* by employing strict pasture management, as well as stable hygiene and farm management. For example, regular removal of manure from the pastures and systematic rotation of horses in paddocks and pens is recommended [49]. Yearly coprological examination is recommended to assess the quality and intensity of infection, as well as the efficacy of the management procedures [7].

## Conclusions

The results of the FECRT with PYR revealed conclusive evidence of the presence of resistant cyathostomins on five farms (19%). As only very few horses were found to shed eggs 14 days after treatment, the efficacy of IVM was found to be very high on the Lithuanian horse farms studied. While resistance to IVM was not detected and PYR is not yet prevalent, the correct use of antihelmintic drug applications and continued monitoring of efficacy with FECRT is important for slowing down the development AR in equine gastrointestinal parasites.

## Supplementary Information

**Additional file 1.** The mean pre-treatment and post-treatment faecal egg count, faecal egg count reduction percentage, and lower and upper 95% confidence limits for each group of anthelmintic drugs tested.

## Data Availability

The datasets used and/or analysed during the current study are available from the corresponding author on reasonable request.
