# Supplementary material for: Anthelmintic resistance of horse strongyle nematodes to ivermectin and pyrantel in Lithuania
Source: Acta Vet Scand. 2021 Jan 25;63:5. doi: 10.1186/s13028-021-00569-z (PMC7836172; doi:10.1186/s13028-021-00569-z)
Supplement: Supplementary file 1 — Additional file 1. The mean pre-treatment and post-treatment faecal egg count, faecal egg count reduction percentage, and lower and upper 95% confidence limits for each group of anthelmintic drugs tested. [file 13028_2021_569_MOESM1_ESM.docx]

**Additional file 1:** The mean pre-treatment and post-treatment faecal egg count, faecal egg count reduction percentage, and lower and upper 95% confidence limits for each group of anthelmintic drugs tested

| **Farm no.** | **Sample size** | **Treatment group** | **EPG at D0 mean** ± **SD** | **EPG at D14 mean** ± **SD** | **FECR%** | **LCL** | **UCL** |
| --- | --- | --- | --- | --- | --- | --- | --- |
| 1 | 10 | IVM | 480 ±146.0 | 0** | 100 | - | - |
|  | 10 | PYR | 369 ±120.2 | 0** | 100 | - | - |
|  | 5 | Control | 464 ±176.3 | 536±140.9 | NA | NA | NA |
| 2 | 10 | IVM | 566 ±406.8 | 2 ±6.33* | 99.9 | 99.5 | 100 |
|  | 10 | PYR | 520 ±321.0 | 81±119.3* | 88.3¹ | 71.7¹ | 100¹ |
|  | 15 | Control | 558 ±241.8 | 583±173.8 | NA | NA | NA |
| 3 | 10 | IVM | 826 ±649.9 | 0** | 100 | - | - |
|  | 10 | PYR | 942 ±618.9 | 38 ±47.6* | 95.9 | 93.9 | 100 |
|  | 5 | Control | 452 ±121.3 | 496 ±95.3 | NA | NA | NA |
| 4 | 10 | IVM | 464 ±215.4 | 0** | 100 | - | - |
|  | 10 | PYR | 397 ±143.3 | 6 ±18.9 | 98.5 | 96.2 | 100 |
|  | 7 | Control | 663 ±198.9 | 710 ±215.7** | NA | NA | NA |
| 5 | 10 | IVM | 890 ±601.1 | 0** | 100 | - | - |
|  | 10 | PYR | 1037 ±811.6 | 126 ±142.4** | 91.6 | 83.1 | 98.7 |
|  | 10 | Control | 719 ±502.3 | 735 ±499.9** | NA | NA | NA |
| 6 | 10 | IVM | 290 ±106.4 | 0** | 100 | - | - |
|  | 10 | PYR | 320 ±171.0 | 18 ±38.2* | 97.2 | 89.2 | 100 |
|  | 5 | Control | 276 ±99.4 | 636 ±226.0 | NA | NA | NA |
| 7 | 10 | IVM | 2021 ±1529.3 | 0** | 100 | - | - |
|  | 10 | PYR | 466 ±224.1 | 36 ±63.1 | 94.8 | 86.2 | 100 |
|  | 13 | Control | 536 ±276.9 | 760 ±194.4* | NA | NA | NA |
| 8 | 10 | IVM | 486 ±312.3 | 0** | 100 | - | - |
|  | 10 | PYR | 422 ±191.5 | 40 ±75.4** | 94.8 | 86.2 | 100 |
|  | 5 | Control | 580 ±226.3 | 720 ±242.1* | NA | NA | NA |
| 9 | 10 | IVM | 2044 ±1701.4 | 0** | 100 | - | - |
|  | 10 | PYR | 1048 ±1241.6 | 140 ±169.4** | 86.4¹ | 79.8¹ | 98.1¹ |
|  | 15 | Control | 725 ±1294.8 | 844 ±415.3** | NA | NA | NA |
| 10 | 10 | IVM | 1104 ±1401.2 | 0** | 100 | - | - |
|  | 10 | PYR | 506 ±352.8 | 6 ±13.5 | 99.4 | 97.6 | 100 |
|  | 5 | Control | 680 ±193.9 | 784 ±153.2* | NA | NA | NA |
| 11 | 10 | IVM | 836 ±410.4 | 0** | 100 | - | - |
|  | 10 | PYR | 1278 ±843.6 | 314 ±326.2** | 75.4¹ | 70.5¹ | 90.5¹ |
|  | 13 | Control | 697 ±281.8 | 917 ±274.3** | NA | NA | NA |
| 12 | 10 | IVM | 452 ±297.7 | 0** | 100 | - | - |
|  | 10 | PYR | 392 ±242.4 | 10 ±25.4 | 97.4 | 94.7 | 100 |
|  | 5 | Control | 424 ±338.1 | 692 ±270.0 | NA | NA | NA |
| 13 | 10 | IVM | 782 ±211.3 | 0** | 100 | - | - |
|  | 10 | PYR | 1010 ±725.6 | 78 ±82.9* | 92.7 | 89.1 | 98.5 |
|  | 10 | Control | 520 ±256.9 | 580 ±225.7** | NA | NA | NA |
| 14 | 10 | IVM | 291 ±167.2 | 0** | 100 | - | - |
|  | 10 | PYR | 700 ±404.3 | 18 ±57.7 | 97.4 | 90.4 | 100 |
|  | 8 | Control | 497 ±252.8 | 817 ±305.6 | NA | NA | NA |
| 15 | 10 | IVM | 1594 ±1635.4 | 0** | 100 | - | - |
|  | 10 | PYR | 1026 ±645.5 | 92 ±120.1** | 91.0 | 88.0 | 99.6 |
|  | 6 | Control | 670 ±517.03 | 697 ±487.0** | NA | NA | NA |
| 16 | 10 | IVM | 468 ±227.9 | 0** | 100 | - | - |
|  | 10 | PYR | 720 ±373.3 | 42 ±94.9 | 92.7 | 82.5 | 100 |
|  | 5 | Control | 780 ±696.8 | 912 ±710.8** | NA | NA | NA |
| 17 | 10 | IVM | 2044 ±2014.3 | 2 ±6 | 99.9 | 99.7 | 100 |
|  | 10 | PYR | 810 ±1091.0 | 78 ±92.1** | 91.6 | 83.9 | 97.9 |
|  | 6 | Control | 1332 ±923.7 | 1348 ±915.7* | NA | NA | NA |
| 18 | 10 | IVM | 408 ±214.2 | 0** | 100 | - | - |
|  | 10 | PYR | 634 ±309.8 | 122 ±157.0** | 87.2¹ | 75.1¹ | 98.1¹ |
|  | 5 | Control | 410 ±221.7 | 536 ±212.3** | NA | NA | NA |
| 19 | 10 | IVM | 790 ±538.1 | 0** | 100 | - | - |
|  | 10 | PYR | 1002 ±1069.5 | 232 ±161.9* | 71.8¹ | 57.6¹ | 85.8¹ |
|  | 7 | Control | 743 ±232.0 | 880 ±298.2** | NA | NA | NA |
| 20 | 10 | IVM | 336 ±96.9 | 0** | 100 | - | - |
|  | 10 | PYR | 378 ±153.3 | 0** | 100 | - | - |
|  | 15 | Control | 508 ±242.3 | 533 ±235.9** | NA | NA | NA |
| 21 | 10 | IVM | 368 ±209.6 | 2 ±6.3 | 99.7 | 98.6 | 100 |
|  | 10 | PYR | 1448 ±1039.9 | 134 ±161.7** | 90.6 | 76.5 | 100 |
|  | 8 | Control | 997 ±747.5 | 1100 ±721.1* | NA | NA | NA |
| 22 | 10 | IVM | 604 ±289.5 | 0** | 100 | - | - |
|  | 10 | PYR | 622 ±373.4 | 44 ±69.2** | 98.5 | 85 | 100 |
|  | 5 | Control | 716 ±558.1 | 848 ±559.0 | NA | NA | NA |
| 23 | 10 | IVM | 278 ±80.0 | 0** | 100 | - | - |
|  | 10 | PYR | 433 ±234.9 | 34 ±47.2* | 94.8 | 89.6 | 99.4 |
|  | 10 | Control | 377 ±190.2 | 634 ±335.9 | NA | NA | NA |
| 24 | 10 | IVM | 1026 ±878.9 | 0** | 100 | - | - |
|  | 10 | PYR | 780 ±654.3 | 8 ±13.9 | 99.3 | 97.8 | 100 |
|  | 11 | Control | 567 ±215.4 | 732 ±239.5** | NA | NA | NA |
| 25 | 10 | IVM | 758 ±568.7 | 0** | 100 | - | - |
|  | 10 | PYR | 566 ±292.4 | 0** | 100 | - | - |
|  | 8 | Control | 716 ±310.3 | 860 ±369.8** | NA | NA | NA |

SD: standard deviation, NA: not applicable, D0: day 0 (treatment day), and D14: day 14 (after treatment), IVM: ivermectin, PYR: pyrantel, FECR%: faecal egg count reduction, LCL: lower confidence level, UCL: upper confidence level; ¹ indicate resistance. Statistically different at *P < 0.05, **P < 0.01.
